# Enzymatic Interesterification of Coconut and Hemp Oil Mixtures to Obtain Modified Structured Lipids

**DOI:** 10.3390/foods13172722

**Published:** 2024-08-28

**Authors:** Ranahansi Rangadharee Bandara, Chloé Louis-Gavet, Joanna Bryś, Diana Mańko-Jurkowska, Agnieszka Górska, Rita Brzezińska, Marta Siol, Sina Makouie, Bharani Kumar Palani, Marko Obranović, Piotr Koczoń

**Affiliations:** 1 Coconut Processing Research Division, Coconut Research Institute, Lunuwila 61150, Sri Lanka; ranahansibandara@gmail.com; 2 CPE-Lyon (École Supérieure de Chimie, Physique, Électronique de Lyon), 43 Boulevard du 11 Novembre 1918, 69616 Villeurbanne, France; 3 Department of Chemistry, Institute of Food Sciences, Warsaw University of Life Sciences, Nowoursynowska st. 159c, 02-787 Warsaw, Poland; diana_manko-jurkowska@sggw.edu.pl (D.M.-J.); agnieszka_gorska@sggw.edu.pl (A.G.); rita_brzezinska@sggw.edu.pl (R.B.); marta_siol@sggw.edu.pl (M.S.); sina_makouei@sggw.edu.pl (S.M.); bharani_palani@sggw.edu.pl (B.K.P.); piotr_koczon@sggw.edu.pl (P.K.); 4 Department of Food Engineering, Faculty of Food Technology and Biotechnology, University of Zagreb, Pierottijeva 6, 10000 Zagreb, Croatia; mobran@pbf.hr

**Keywords:** coconut oil, hemp oil, interesterification, fatty acid composition, oxidative stability

## Abstract

The interesterification process allows structured lipids (SLs) to be obtained with a modified triacylglycerol (TAG) structure, in which the unfavorable saturated fatty acids (SFAs) are replaced with nutritionally significant fatty acids (FAs) such as monounsaturated (MUFAs) and polyunsaturated (PUFAs). Oxidative stability is crucial for the quality of SLs. This study aimed to characterize and evaluate the FA profile and oxidative stability of SLs synthesized by the enzymatic interesterification of hemp seed oil (HO) and coconut oil (CO) blends. Blends were prepared in three ratios (75% HO:25% CO, 50% HO:50% CO, and 25% HO:75% CO) and interesterified using *sn*-1,3 regiospecific lipase for 2 or 6 h. FA composition, the FA distribution of TAGs, acid value (AV), peroxide value (PV), and oxidation time were analyzed and compared to non-interesterified blends. Results showed no significant difference in the SFA:MUFA ratios between interesterified and non-interesterified blends with the same proportions. Lauric acid predominantly occupied the *sn*-2 position in all blends. Interesterified blends had higher AVs, exceeding codex standards, while PVs remained within the acceptable limits. Blends with 75% HO had lower oxidation times compared to those with 75% CO, with no significant difference between interesterified and non-interesterified blends. In the interesterification process of the studied blends, new TAGs with a modified structure were created, which may affect their physical and nutritional properties. This process also had a significant effect on the AV and PV levels, but not on the oxidation time of the modified blends. Therefore, it is necessary to remove free FAs after the enzymatic process to produce SLs characterized by improved hydrolytic stability. This will lead to better technological properties compared to the original oils. Further research is also necessary to enhance the oxidation stability of SLs obtained from blends of CO and HO to improve their storage stability.

## 1. Introduction

Natural oils and fats have a significant role to play in human nutrition as they are a vital source of energy and essential FAs. Additionally, they act as carriers for fat-soluble vitamins. Triacylglycerols (TAGs) constitute the majority of these oils and fats. The molecular composition and structure of TAGs play a crucial role in determining their physicochemical as well as their functional and nutritional properties [1,2]. The source of the lipid determines the proportion of each type of FA [3].

Blending and interesterification are two widely used techniques in the edible oil industry to enhance the physiochemical and functional properties of oils. Blending involves mixing different oils to achieve specific FA compositions and textures. However, the major limitation of blended products is that it can result in phase separation issues when oils with different melting points are combined. Interesterification is a chemical or enzymatic process that utilizes a catalyst to rearrange fatty acids within and between TAG molecules, resulting in SL modified lipids that exhibit different physical and chemical characteristics. This process leads to the production of trans-free plastic fats and is capable of overcoming the limitations related to blended products [4,5].

The process of interesterification can be either chemical or enzymatic. The use of chemical catalysts allows TAGs to be obtained with a changed structure, but this process requires the use of potentially toxic substances, and by-products and waste may be generated during this process, therefore it is not beneficial for the environment. However, enzymatic interesterification is a more desirable method compared to chemical interesterification for several reasons. Firstly, it involves mild processing conditions, which is advantageous. The use of enzymes with different specificities allows lipids to be obtained with pre-planned properties that cannot be obtained by chemical modification. Moreover, this method produces fewer by-products and provides easy control over the process. Therefore, enzymatic interesterification is considered as a green technology that generates modified lipids with enhanced functional and nutritional properties and is free of *trans* fats. The SLs could be implemented in the food industry as well as in clinical nutrition [3,6,7,8].

Coconut (*Cocos nucifera* L.), which belongs to the family Arecaceae, is a versatile and widely used ingredient in Asian cuisines while Indonesia, the Philippines, India, and Sri Lanka are considered the major commercial cultivators [9]. The coconut oil (CO) produced by pressing the coconut kernel is primarily comprised of 92% saturated fats, with over 50% of it coming from medium-chain fatty acids (MCFAs) such as C8:0, C10:0, and C12:0 [10]. The consumption of SFAs has long been linked to an increase in low-density lipoprotein (LDL) cholesterol levels, and therefore, an increased risk of developing cardiovascular and other heart-related diseases [11]. Nevertheless, research indicates that various SFAs have different effects on LDL cholesterol, high-density lipoprotein (HDL) cholesterol, and the total cholesterol (TC)/HDL ratio. Lauric acid (12:0) can raise LDL cholesterol higher than myristic (14:0) and palmitic (16:0) acids [12]. Consequently, replacing some of the SFAs like lauric acid with nutritionally significant long-chain fatty acids (LCFAs) such as MUFAs and PUFAs would provide better health outcomes.

Hemp (*Cannabis sativa* L.) is an herbaceous plant that has been cultivated for centuries for its fiber and oil. Hemp seeds contain a high percentage (25–35%) of polyunsaturated oil that is rich in essential fatty acids (EFAs). Linoleic acid, an ω-6 acid, is the main FA, while α-linolenic acid, an ω-3 acid, is the second most abundant FA present in hemp oil seeds. Additionally, HO contains approximately 1–5% γ-linolenic acid, which is rarely found in seed oils. The ratio of the two essential PUFAs (linoleic and α-linolenic acids) in HO is 3:1, making it a perfectly balanced source of nutrition for human consumption [13,14]. HO has been a valuable source for the food and pharmaceutical industries since ancient times due to its high content (more than 80%) of PUFAs [15,16]. Therefore, enzymatic interesterification can be used to incorporate the beneficial FAs from HO into CO.

Oxidative stability is a crucial indicator that determines the quality of SLs [17]. The oxidation of PUFAs leads to the formation of free radicals, hydroperoxides, and finally short-chain aldehydes and ketones, which can reduce the storage stability and quality of fats and oils. The oxidation of fats and oils during storage produces distinct off-flavors and odors. The oxidative stability of modified fats and oils is influenced by various factors such as production methods (e.g., chemical, enzymatic, etc.), purification methods (e.g., alkaline, deodorization, distillation, etc.), oil sources, and the presence of antioxidants during production. Furthermore, the stability of structured fats and oils is influenced by various parameters such as the molecular structure of TAGs including the FA composition, positional distribution of FAs on the glycerol backbone, and the interaction of both factors [3,18]. Although previous studies have been carried out on the enzymatic interesterification of CO [8,19,20] and HO [13,21] separately with other edible fat sources, studies on the enzymatic interesterification of blends of coconut oil and HO are still lacking in the literature. Consequently, to the best of our knowledge, there are currently no reports available on the assessment of the oxidative stability of interesterified coconut and hemp seed oils.

Considering that previously stated, this study aimed to evaluate the FA profile and the oxidative stability of SLs synthesized by enzymatic interesterification (using *sn*-1,3 regiospecific lipase) of the blend of HO with CO. It has been predicted that lipases with *sn*-1,3 regiospecificity are beneficial in the production of SLs [22]. Accordingly, the research bridges a significant gap in the existing literature by investigating the potential of enzymatic interesterification in creating SLs that possess a well-balanced FA composition and modified nutritional and functional properties using two edible vegetable oils. Moreover, this study can contribute to the advancement of the food and pharmaceutical industries by exploring the feasibility of this process and can promote the development of healthier and more functional products.

## 2. Materials and Methods

### 2.1. Materials and Chemicals

All solvents and reagents of analytical grade were obtained from Avantor Performance Materials Poland S.A. (Gliwice, Poland), except for the porcine pancreatic lipase (Type II), which was sourced from Sigma-Aldrich (Saint Louis, MO, USA) along with the standard compounds. The immobilized Lipozyme RM IM (enzymatic catalyst) is produced by the immobilization of a lipase from *Rhizomucor miehei* fungi on a macroporous anion exchange resin and shows regiospecific activity against ester bonds at the *sn*-1,3 position of the triacylglycerol backbone. This enzymatic catalyst was also acquired from Sigma-Aldrich. The silica gel TLC plate was purchased from Merck KGaA (Darmstadt, Germany). The HO and CO were provided by a commercial supplier.

### 2.2. Enzymatic Interesterification

Based on previous studies [7,13], the initial oil blends (20 g) were prepared in three different weight ratios (75% HO:25% CO, 50% HO:50% CO, and 25% HO:75% CO). The oil mixtures were placed in flasks and placed in a temperature-controlled mineral oil bath shaker. Once the samples reached a temperature of 60 °C (about 5 min), an enzymatic catalyst (8% *w*/*w*) was added to start the enzymatic interesterification. The interesterification reactions were carried out in an Elpin Plus type 357 laboratory shaker (Lubawa, Poland) for 2 h or 6 h with simultaneous temperature control (60 °C) and continuous agitation (100–150 rpm). To complete the reaction, the enzyme catalyst was separated from the reaction products by filtration under vacuum in a Büchner funnel. Then, the obtained oil samples were transferred to plastic screw-cap containers and maintained for a maximum of 1–2 days at −18 °C for further analysis. In order to determine the composition and structure of the TAGs, the TAG fraction had to be isolated by column chromatography on silica gel (SG 60, 70–230 mesh, Merck, Germany) with a mixture of petroleum ether:ethyl ether (87:13 v/v), which was evaporated after column chromatography.

### 2.3. Determination of Fatty Acid Composition

The determination of FA composition was carried out by gas chromatographic (GC) analysis of the fatty acid methyl esters (FAMEs). GC was chosen for FA composition determination because it offers a combination of high sensitivity, specificity, and the ability to quantify a wide range of FAs with great accuracy and precision. FAMEs were prepared through transesterification with sodium methoxide according to ISO 5509:2001 [23]. A YL6100 GC chromatograph equipped with a flame ionization detector and BPX-70 capillary column (60 m length, 0.25 µm film thickness, and 0.25 mm internal diameter) was used. The oven temperature was programmed as follows: 70 °C for 30 s, and then it was increased by 15 °C min^−1^ to 160 °C; from 160 to 200 °C, it was increased by 1.1 °C min^−1^, and then kept at 200 °C for 12 min, and next from 200 to 225 °C, it was increased by 30 °C min^−1^, and then kept at 225 °C for 60 s. The temperature of the injector was 225 °C, with a split ratio of 1:50 and the detector temperature was 250 °C. Nitrogen flow with the rate of 1 mL min^−1^ was used as the carrier gas. The results were expressed as relative percentages of each FA (% of the FA peaks area was calculated). FAs were identified by comparing the relative retention times of the FAME peaks with the FAME chemical standard (Supelco 37 Component FAME Mix).

### 2.4. Determination of Positional Distribution of Fatty Acids on sn-2 and sn-1,3 Positions of TAGs

The distribution of FAs in the *sn*-2 and *sn*-1,3 positions of TAGs was determined by the selectivity of pancreatic lipase in hydrolyzing ester bonds in the *sn*-1,3 positions. To achieve this, 20 mg of purified pancreatic lipase (crude type II, porcine pancreatic lipase), 1 mL of Tris buffer (pH 8.0), 0.25 mL of bile salts (0.05%), and 0.1 mL of calcium chloride (2.2%) were combined in 50 mL centrifuge tubes and mixed with 0.1 g of fat sample using a vortex. The mixture was then incubated at 40 °C in a water bath for 5 min, followed by adding 1 mL of 6 mol L^−1^ HCl and 4 mL of diethyl ether and centrifuging the mixture. The diethyl ether layer was collected in test tubes and evaporated under nitrogen gas until it reached a volume of 200 μL. A 200 μL aliquot was applied onto a silica gel TLC plate with fluorescent indicator 254 nm and developed with hexane/diethyl ether/acetic acid (50:50:1, *v*:*v*:*v*). The *sn*-2 monoacylglycerol band was observed under UV light. The band was removed from the plate and transferred into a screw-capped test tube, where it was extracted twice with 1 mL of diethyl ether and then centrifuged. The ether layer was collected and completely evaporated under nitrogen, and the sample was dissolved in n-hexane and methylated.

The GC method was used to analyze the FA composition of the *sn*-2 monoacylglycerols obtained. The FA composition in the *sn*-1,3 positions was determined by comparing the compositions of the isolated *sn*-2 monoacylglycerols and the starting TAGs. The following equations were used to make these determinations [24]:

(1) $$sn-1,3=\frac{3\times ({FA}_{in\;TAG})-({FA}_{in\;sn-2\;MAG})}{2}$$

(2) $$sn-2=\frac{({FA}_{in\;sn-2\;MAG})\times 100\%}{3\times \left({FA}_{in\;TAG}\right)}$$

where:

*sn*-1,3 is the content of a given fatty acid in *sn*-1 and *sn*-3 positions [%];
FA in TAG is the content of the given fatty acid in the starting triacylglycerols (TAGs) [%];
FA in *sn*-2 MAG is the content of the given fatty acid in *sn*-2 monoacylglycerols (MAGs) [%].

### 2.5. Determination of Acid Value

The acid value (AV) was determined by the titration of fat samples with 0.1 M ethanolic potassium hydroxide solution following the AOCS method (AOCS Official Method Te 1a-64) [25].

### 2.6. Determination of Peroxide Value

The peroxide value (PV) of the mixture was determined by the iodometric technique with visual endpoint detection in accordance with the AOCS method (AOCS Cd 8b-90) [26].

### 2.7. Determination of Oxidation Time

The analysis was performed using a DSC Q20 TA instrument coupled with a high-pressure cell (PDSC). Fat samples weighing 3–4 mg were placed in an aluminum open pan and introduced into the sample chamber at an initial oxygen pressure of 1400 kPa. The isothermal temperature for each sample was set at 120 °C. Data obtained were analyzed using TA Universal Analysis 2000 software and the output was automatically recalculated and presented as energy per gram. The maximum PDSC oxidation time (induction time) was determined based on the maximum rate of oxidation.

### 2.8. Statistical Analysis

Data analysis was performed using the Minitab statistical software. One-way ANOVA and factorial designs were used as appropriate. The mean separation was carried out at a *p*-value of 0.05, according to Tukey’s multiple range test. All analyses were performed in triplicate.

## 3. Results and Discussion

### 3.1. Fatty Acid Composition of Oil Samples

**Figure 1:**
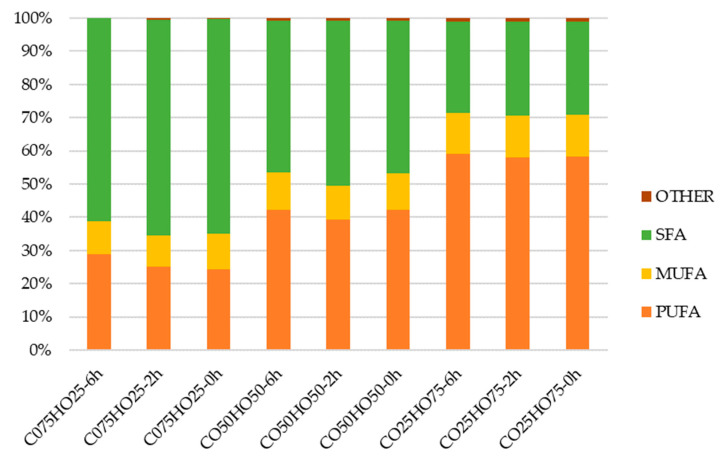
Fatty acid composition (%) of the coconut oil (CO) and hemp oil (HO) blends with different oil ratios without interesterification and after 2 or 6 h of interesterification, where SFA—saturated fatty acid, MUFA—monounsaturated fatty acid, PUFA—polyunsaturated fatty acid. The different lowercase letters (for each group of fatty acids separately) indicate significantly different values (*p* ≤ 0.05). Data are presented as mean values.

Based on the data presented in Figure 1, it was found that the FA profiles of the interesterification products remained unchanged, but the FA composition of the blended oils was altered based on the proportion of each oil type used. This means that the ratio of SFA to MUFA to PUFA varied depending on the specific blend of oils used.

According to the analysis, linoleic acid (52.7–52.8%) was found to be the most significant PUFA in the HO samples. This FA belongs to the ω-6 acid family. Another important PUFA found in the samples was α-linolenic (16.3%), belonging to the ω-3 family. The analyzed HO samples also contained γ-linolenic acid, in the amount of 4.6–4.7%. The CO samples used in the study were composed of 90.85% of SFA including 42.86–43.29% of lauric acid, 19.58–19.76% myristic acid, and 9.41–9.73% palmitic acid. The results were consistent with previous studies conducted by Bryś et al. [27] on HO and Sivakanthan et al. [28] on the enzymatic interesterification of coconut oil. The ratio of ω-6 to ω-3 acids was found in HO to be approximately 3:1, which is very beneficial from a nutritional perspective. Such a ratio may have the effect of lowering the level of bad cholesterol in the blood (LDL—low-density lipoproteins) as well as lowering blood pressure. The presence of linoleic and α-linolenic acids is beneficial for consumers, especially for those who are looking for food or a diet that provides antiarrhythmic, anticoagulant, or anti-inflammatory effects. The nutritional value of HO is additionally enhanced by the presence of γ-linolenic acid, belonging to the ω-6 fatty acids. It also reveals a pharmacological effect, among others, in regulating the body’s inflammatory responses. γ-Linolenic acid is a rather rare acid not found in the popularly used vegetable oils [29]. CO consists of MCFAs, primarily lauric acid. Its metabolic pathways differ from those of long-chain TAGs, resulting in numerous health benefits. Consuming MCFAs can reduce body fat levels and lower the risk of heart disease and diabetes. Moreover, CO supplementation has been found to play a crucial role in cancer prevention and treatment as well as in reducing the biological activity of various pathogenic bacteria and viruses [30]. Accordingly, interesterified blends of HO and CO may yield a more stable oil complex with combined health benefits derived from the FAs present in both types of oils.

The study found that even after the interesterification, the level of SFAs was significantly high in oil blends with a high amount of CO, while the level of PUFAs was correlated with the amount of HO present, due to the high concentration of PUFA in HO (74.37%) and SFA in CO (90.85%).

Even though PUFAs in dietary lipids are essential in helping to decrease the serum cholesterol concentration, their consumption in excessive amounts results in exerting oxidative stress [31]. According to the recommendations of the Indian Council of Medical Research and the American Heart Association, it is desirable to consume oils with equal amounts (1:1:1) of SFA, MUFA, and PUFA. The World Health Organization recommends a ratio of 1:1.5:1 for SFA, MUFA, and PUFA. However, any of the oils in their natural form do not fulfill this requirement. Therefore, the development of modified lipids is crucial for a healthier consumption pattern of fats and oils [32]. According to the results of the present study, 100% of HO contained 74.37% of PUFA, 13.5% of MUFA, and 10.6% of SFA. The blending and interesterification of HO with CO in the ratio of 1:1 resulted in a modified lipid with nearly 40–50% PUFA and SFA but the MUFA remained around 10–11%. It was found that there was no significant difference between the SFA:MUFA:PUFA ratio in the interesterified and non-interesterified oil blends when they had the same oil proportion. According to the results of the study, it was observed that there is potential for balancing the 1:1:1 ratio of SFA:MUFA:PUFA through the blending and interesterification of the oils, even though a perfect rearrangement of FA on their triglyceride moieties was not achieved through the interesterification in the present study. Blending vegetable oils, which are high in unstable FAs—PUFA—with more stable CO (high in SFA), can increase their thermal and oxidative stability, making them more suitable for cooking [33].

### 3.2. Positional Distribution of Fatty Acids on sn-2 and sn-1,3 Positions of Triacylglycerols

The physical and chemical properties of fats and oils are primarily determined by the types of FAs present and their positioning within the TAG molecule. Fats and oils containing a higher proportion of SFA in their TAG molecules have elevated melting points, whereas those with a higher proportion of unsaturated fatty acids have lower melting points. Additionally, an increased level of unsaturation enhances the plasticity of fats. Research has indicated that mechanical manipulation and the addition of edible oil can enhance the plasticity and shortening power of fats [34]. Accordingly, the process of blending and interesterification can lead to changes in the FA composition and their distribution in the TAG of both CO and HO. These alterations may contribute to an enhancement in the physiochemical properties of the oils compared to their pure forms.

The results of the FA composition at the *sn*-2 (internal) and *sn*-1,3 (external) TAG positions of the interesterified and non-esterified oil blends of coconut and hemp seed are represented in Table 1.

TAGs consist of a glycerol backbone bonded to three esterified FAs and form the main components of vegetable oils. The positional distribution of FAs in the *sn*-2 and *sn*-1,3 positions of TAG is based on the ability of the pancreatic lipase to selectively hydrolyze ester bonds in the *sn*-1,3 positions. The structure and arrangement of TAGs play a vital role in lipid metabolism. Furthermore, the structural configuration of TAGs influences the physical properties of fats such as the melting point, solid fat content, crystal structure, and susceptibility to oxidation and polymerization [35].

In accordance with the results, the interesterified oil blends of CO and HO showed variations in the distribution of FAs within the TAG molecule, with different acids occupying the inner and outer positions. The *sn*-2 position was predominantly occupied by lauric acid (C12:0), with each blend constituting over 33% of the equilibrium share, regardless of the oil ratios and interesterification duration. Based on the analysis of SFAs including myristic acid (C14:0), palmitic acid (C16:0), and stearic acid (C18:0), it was observed that after interesterification, myristic acid exhibited an FA share of more than 33% on the *sn*-2 position. This was achieved with the 25% of CO and 75% of HO and with 50% of CO and 50% of HO blends, both interesterified for 6 h. In contrast, the other SFAs were predominantly distributed in the external positions of TAGs, with their share in the *sn*-2 position being less than 33%. Furthermore, when SFAs were esterified in the *sn*-1 and *sn*-3 positions, their digestibility was lower compared to unsaturated FAs in the same position [24]. According to the analysis, lauric acid (C12:0) and oleic acid (C18:1) were mainly present in the internal position of TAGs from CO, while the external position was preferred by myristic acid (C14:0), palmitic acid (C16:0), and stearic acid (C18:0). However, when it comes to HO, the internal *sn*-2 position of the TAGs of this oil was mainly occupied by unsaturated fatty acids (i.e., oleic acid (C18:1), linoleic acid (C18:2), and α-linolenic acid (C18:3)).

In the analysis of unsaturated FAs in oil blends subjected to a 2-h interesterification process, it was observed that oleic acid (C18:1 n-9) and linoleic acid (C18:2 n-6) accounted for over 33% of the *sn*-2 position, while other blends mostly had a lower percentage. Furthermore, α-linolenic acid (C18:3 n-3) was found to exceed 33% after 2 h of enzymatic interesterification in oil blends containing 25% CO and 75% HO, 50% CO and 50% HO as well as in oil blends subjected to 6 h of interesterification with 50% CO and 50% HO and 75% CO and 25% HO. According to previous research on plant oil blends, lauric acids were always present in the internal positions of TAGs, whereas palmitic acid was present in the external positions of TAGs, which is consistent with our findings [35]. TAGs rich in palmitic acid at the *sn*-2 position and unsaturated FAs at the *sn*-1,3 position of the glycerol backbone play a crucial role as a source of nutrients and energy for human metabolism [36]. It has been noted that interesterification resulted in a significant increase in the distribution of palmitic acid in the *sn*-2 position after 6 h, although the percentage was lower than the average of 33%. Additionally, in the 6-h interesterification process using a blend of 75% CO and 25% HO, there was a significant increase in the distribution of oleic acid, linoleic, and α-linolenic acid in the *sn*-2 position compared to the non-interesterified oil blend.

### 3.3. Acid Value of Oil Samples

**Figure 2:**
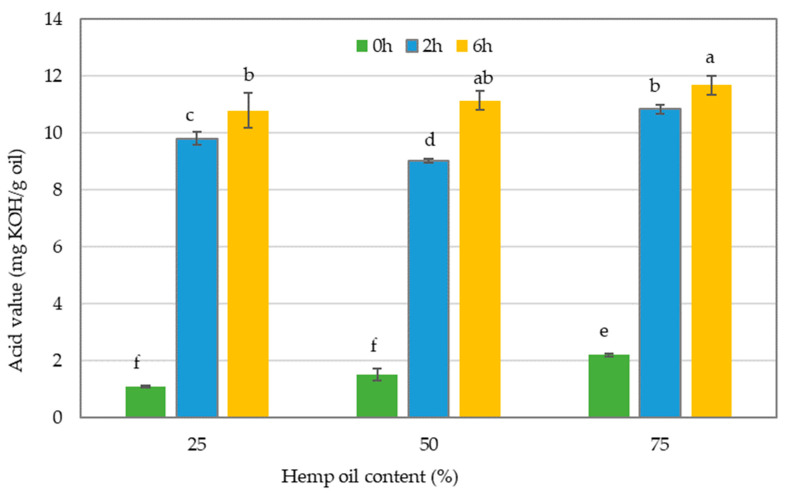
The acid value of oil blends without interesterification and after 2 or 6 h of interesterification. The different lowercase letters indicate significantly different values (*p* ≤ 0.05). Data are presented as mean values followed by the standard deviation (±SD).

The AV is a crucial parameter that determines the quality of vegetable oils. It is measured by the quantity of potassium hydroxide in milligrams required to neutralize one gram of oil. This value is an essential criterion for determining the freshness and oxidative stability of the oil, which can impact its shelf life and suitability for various applications [37]. The AV of the oil blends calculated for different hours of interesterification is represented in Figure 2.

During the interesterification process, which involves the exchange of FAs between two glyceride molecules, changes in AV are inevitable due to the production of free fatty acids (FFAs) and partial acylglycerols [38]. According to the CODEX standards, the maximum permissible level of AV for cold-pressed vegetable oils is 4.0 mg KOH/g of oil [39]. The acid value, which is a measure of the amount of FFAs in the oil, was for HO and CO 2.33 and 0.67 mg KOH/g, respectively, which is a value in accordance with the codex standard. Additionally, as represented in Figure 2, the AV of the three oil blends that were not interesterified (0 h) and had no enzyme involvement was found to be below the maximum permissible limit under the codex standards. However, the AV of the oil blends that underwent enzymatic interesterification was significantly higher compared to those that did not undergo the process and were above the codex standard. The presence of enzymes and water in the reaction mixture led to elevated levels of FFA contents in the oils [40]. In the process of interesterification, lipases and high temperatures can cause the breakdown of oils and the formation of FFAs. This could lead to an increase in the AV of the oil samples [41]. Yazdi and Alemzadeh [42] also indicated that an AV of 0.065–0.07 mg KOH/g of oil in blends of palm oil and sunflower oil before interesterification was raised between 2.52 and 4.32 mg KOH/g of oil after interesterification following an increasing trend, as observed in the present study. However, FFAs are prone to oxidation and their high levels can lead to the decreased oxidation stability of structured fats, causing flavor and color deterioration [40]. The use of ethanol to rinse oil blends is an efficient method for reducing the AV of interesterified oil blends [43]. Moreover, as stated by Sivakanthan et al. [8], the amount of FFAs generated during interesterification reactions can be reduced by replacing aqueous enzymes with immobilized enzymes.

**Figure 3:**
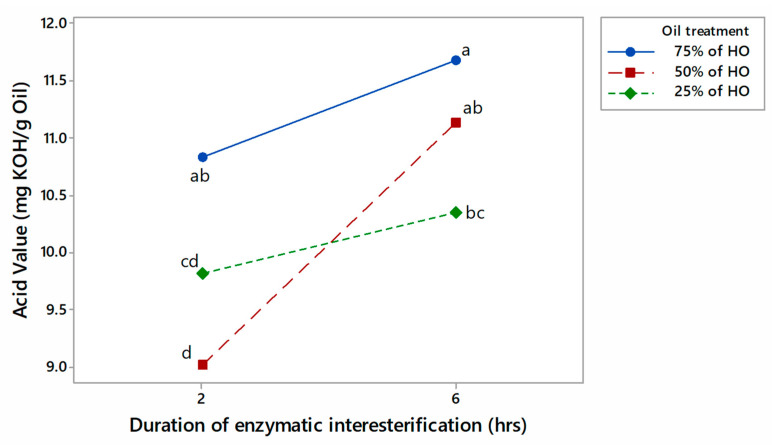
Interaction plot for the acid value of the blend samples (two-factor factorial, means denoted by distinct letters are significantly different).

Furthermore, this study examined the impact of interesterification time and the proportion of different oils in a blend on the AV of the resulting oil. The results (Figure 3) showed that the AV decreased significantly when 50% HO was used in the blend, at 2 h of interesterification compared to 6 h. In contrast, 6 h of interesterification resulted in higher AV, regardless of the proportion of each oil in the blend. Both the 25% HO integrated interesterified oil blend and the 75% HO integrated interesterified oil blend did not show a significant difference in their AV due to changes in the duration of interesterification.

Our findings revealed that the interaction effect of these two factors significantly impacts the AV of the oils (*p* < 0.05) (Figure 3).

Increasing the HO proportion to 75% in the blend led to a significant enhancement in the AV in both the 2-h and 6-h interesterification times compared to increasing the CO to 75%. The higher AV of the interesterified blends containing more HO may be related to the higher AV of the raw material HO compared to CO. A higher AV is associated with a higher content of FFAs, which have lower oxidative stability than the same fatty acids in TAGs. Additionally, a large amount of PUFA in interesterified mixtures containing 75% HO may cause greater oxidative instability [44].

Additionally, our results indicate that 50% of HO with 2 h of interesterification and 25% of HO with 2 h of interesterification did not show a significant difference in their AV. Furthermore, the SLs resulting from 50% of HO with 2 h of interesterification had the lowest AV, which was significantly lower than all the other combinations except 25% of HO with 2 h of interesterification. As a result, the treatment combination that produced SLs with a significantly lower AV using HO and CO was identified as the blend with 50% of HO that underwent 2 h of interesterification. However, in this study, all of the resulting blends showed higher AV than the limits set by the CODEX standards, leading to a lower oxidation stability compared to the non-interesterified oil blends.

The obtained SLs must be purified from FFAs to be used in practical applications. The extraction of FFAs from oils can be achieved through physical and chemical methods. Chemical methods can result in notable oil loss due to saponification and emulsification, whereas physical methods entail high power consumption, although they are more suitable for vegetable oil FFA removal. Therefore, alternative methods such as chemical esterification, membrane technology, and stripping techniques have been proposed in the literature to address these drawbacks and offer more efficient solutions [45]. Accordingly, the utilization of innovative techniques to extract the FFA components from SLs will play a crucial role in ensuring that the AV of SLs remains within the acceptable limits outlined in the codex standard.

### 3.4. Peroxide Value

**Figure 4:**
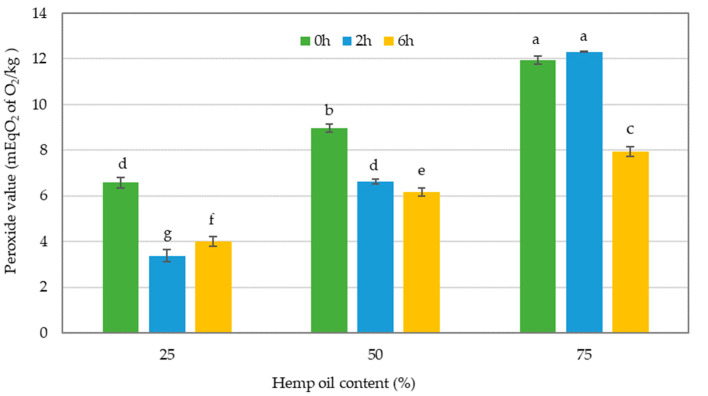
Peroxide value of the blend oil samples without interesterification and after 2 or 6 h of interesterification. The different lowercase letters indicate significantly different values (*p* ≤ 0.05). Data are presented as mean values followed by the standard deviation (±SD).

The peroxide value (PV) is a quantitative measure of the degree of oxidation in edible oils. It serves as an indicator of the freshness of the oil sample, where a higher PV indicates a higher degree of oxidation and a lower degree of freshness [46]. The measurement of PV for both interesterified and non-interesterified oil blends is illustrated in Figure 4.

The results indicated that there was a considerable reduction in the PV of oil blends that contained 25% and 50% HO after 2 and 6 h of interesterification compared to the non-interesterified blends consisting of the same ratio of HO and CO. However, there was a significant reduction in the PV in oil blends that contained 75% HO, only at 6 h of interesterification compared to the non-interesterified oil blends with the same oil ratio. The PV for HO and CO was 13.82 and 4.12 mEqO_2_/kg, respectively. The presence of higher levels of HO in oil blends has been found to cause a significant increase in the PV of the resulting oils, which is a clear indication of accelerated oxidation. A considerable amount of chlorophyll in hemp seed is extracted during the pressing of hemp seed for oil extraction. As a photosensitive pigment, chlorophyll undergoes photo-oxidation, leading to rancidity and the quality deterioration of HO, which therefore necessitates storage in dark or opaque bottles [15,47].The PV limit for vegetable oils was set at 15 mEqO_2_/kg of oil in accordance with the codex standards [39]. Accordingly, all of the oil sample results from the present study recorded a lower PV compared to the codex limits of vegetable oils.

**Figure 5:**
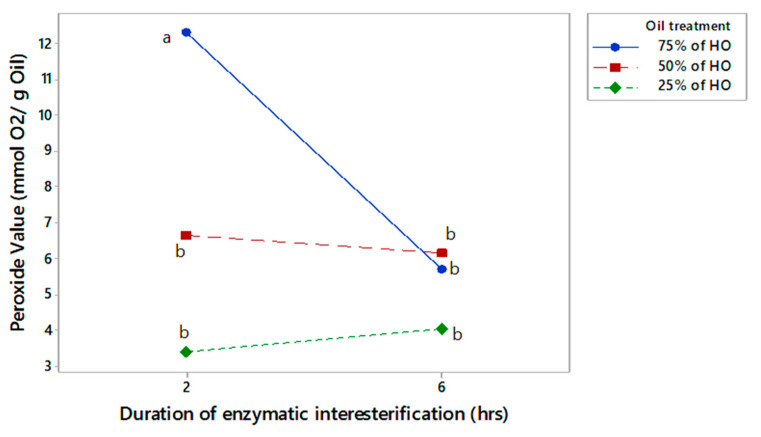
Interaction plot for the acid value of the blended oil samples (two-factor factorial, means denoted by distinct letters are significantly different).

As indicated in the interaction plot presented in Figure 5, the interaction effect of the two factors, the proportion of the two types of oils included in the blend and the duration of enzymatic interesterification, were found to be significant on the PV of the oil blends that underwent enzymatic interesterification. Based on the interaction plot, the oil blend, which comprised 75% HO and was enzymatically interesterified for 2 h, showed a significantly higher PV while the interaction effects of all others were not significantly different from each other. The study findings suggest that a longer interesterification time (enzyme reaction time) can cause a distinct alteration in the arrangement of FAs in TAG molecules, despite the constant concentration of the oil blend. Moreover, the results indicate that for oil blends with a higher PUFA concentration, a longer interesterification time is preferable to reduce the PV for oil blends with higher PUFA concentrations. Yazdi and Alemzadeh [42] reported a reduction in PV after the enzymatic reaction, which supports our findings. The decrease in PV was due to peroxide binding with the enzyme protein. Therefore, our results suggest that prolonging the enzyme reaction time with the oil could further enhance this phenomenon (reaction time) and can cause a distinct alteration in the arrangement of FAs in TAG molecules, despite the constant concentration of the oil blend.

Peroxide levels typically decrease after interesterification due to the binding of peroxides to lipase, resulting in the conversion of peroxides into aldehydes. This binding process leads to the inactivation of the lipase enzyme. The conversion of peroxide to aldehyde causes an increase in the anisidine value and accelerates the spoilage of oil. Therefore, it is crucial to ensure that the initial PV is as low as possible to prevent adverse effects on enzyme activity and to keep the anisidine value within the acceptable range following interesterification [42].

### 3.5. Oxidation Time

In oil stability analysis, the longer the oxidation time, the greater the oxidative stability of the oil. Moreover, the rate of the oxidation process depends on the presence of antioxidants or pro-oxidants [35].

**Figure 6:**
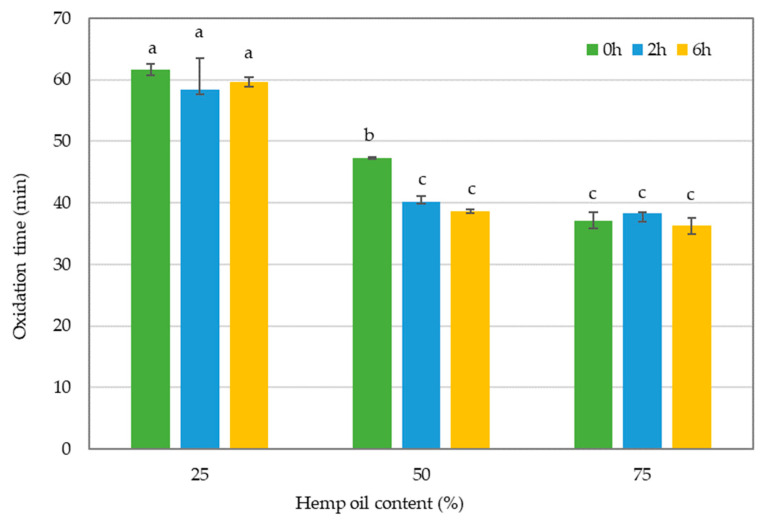
Oxidation time of the oil blend samples without interesterification and after 2 or 6 h of interesterification. The different lowercase letters indicate significantly different values (*p* ≤ 0.05). Data are presented as mean values followed by the standard deviation (±SD).

The study results showed (Figure 6) that oil blends containing 75% HO had a significantly lower oxidation time in comparison to blends with 75% CO. No significant difference was observed between oil blends that underwent enzymatic interesterification and those that did not at these proportions.

HOs are primarily composed of PUFAs (more than 70%), as represented in Figure 1. The majority of PUFAs are from the n-6 and n-3 families [48].

Kowalski et al. [49] concluded that oils and fats containing PUFAs were more susceptible to oxidative changes in their study on the oxidative stability of vegetable oils due to faster oxidation processes. The study found that the sample with the highest HO content (75% HO and 25% CO), which was known to be rich in PUFA, exhibited the lowest oxidative stability. The oxidation time for HO and CO was 30.36 and 72.13 min, respectively. Blends containing a high amount of CO had significantly higher oxidation times, ranging from 55 to 60 min. This was due to the high amount of SFAs in CO, which are more stable against oxidation. These findings are consistent with a study conducted by Brzezińska et al. [35], which showed a similar trend of increased oxidation time with the addition of CO to tomato seed oils for enzymatic interesterification.

A study conducted by Ramezan et al. [50] revealed that the crude CO had a longer oxidation times of about 550 min. However, the present study found that the oxidation stability of crude CO was considerably reduced when interesterified with HO. On the other hand, crude HO, as per Bryś et al. [27], had an oxidation time ranging between 13.6 and 28.9 min. The oxidation time of crude HO increased to 30–40 min by integrating 25% CO and increased to 55–60 min by integrating 75% CO, resulting in higher oxidative stability. The study also proved that the enzymatic reaction (interesterification process) did not affect the oxidation time of oils significantly. Furthermore, the results of the two-factor factorial analysis, conducted only on the interesterified oil blends, showed that the interaction effect of the two factors, enzymatic reaction time and the proportion of each type of oil used in the blend, was not significant for the oxidation time of modified oils (*p* > 0.05).

## 4. Conclusions

Oil blends containing a high proportion of CO following interesterification exhibited elevated levels of SFAs. In contrast, the quantity of PUFAs was associated with HO due to the high concentration of PUFA in HO (74.37%) and SFA in CO (90.85%). However, there was no remarkable disparity in the SFA:MUFA:PUFA ratio between the interesterified and non-interesterified oil blends with the same oil ratio. Lauric acid (C12:0) predominantly occupied the *sn*-2 position, constituting over 33% of the equilibrium share in each blend, regardless of the oil ratios and interesterification duration. The AV of the oil blends subjected to enzymatic interesterification was significantly higher compared to those that did not undergo the process and exceeded the codex standard. The PV of the interesterified and non-interesterified oil blends remained within the maximum limit of the codex standard. Oil blends containing 75% HO exhibited a shorter oxidation time compared to blends with 75% CO. At these proportions, no significant difference was observed between oil blends that underwent enzymatic interesterification and those that did not. The combined impact of two factors (oil proportion and interesterification time) remarkably influenced the acid and peroxide values of the interesterified oil blends, while it had an insignificant effect on the oxidation time. The results of this study have important implications for both the food and pharmaceutical industries. In the food industry, the process of interesterification can be used to create SLs with a balanced FA profile. This can result in the production of healthier cooking oils and food products. Additionally, these SLs can be used to improve the effectiveness of lipid-based pharmaceuticals in treating infections and other health conditions. This study has provided valuable insights into the impact of interesterification on CO and HO blends. However, further research is recommended to enhance the oxidation and hydrolytic stability of these SLs. Future studies should explore the use of natural antioxidants and combinations of oils to improve the storage stability of these blends. Furthermore, research into the optimization of enzymatic interesterification conditions including temperature, enzyme concentration, and reaction time could lead to more efficient and cost-effective processes.

## Figures and Tables

**Table 1:** Fatty acid composition of the outer (*sn*-1,3) and inner (*sn*-2) triacylglycerol (TAG) positions of the coconut oil (CO) and hemp oil (HO) blends with different oil ratios without interesterification and after 2 or 6 h of interesterification.

Fatty Acid	Oil Type	Composition [%]		Distribution [%]
C12:0		TAG	*sn*-1,3	*sn*-2
	CO25HO75 0	8.99 ± 0.21 ^d^	0.64 ± 1.12 ^f^	95.15 ± 8.40 ^a^
	CO25HO75 2	9.26 ± 0.62 ^d^	4.88 ± 1.63 ^de^	65.15 ± 9.37 ^b^
	CO25HO75 6	8.99 ± 0.61 ^d^	3.38 ± 1.07 ^ef^	75.13 ± 6.26 ^b^
	CO50HO50 0	19.21 ± 1.28 ^c^	7.43 ± 2.14 ^d^	74.42 ± 5.72 ^b^
	CO50HO50 2	20.31 ± 0.61 ^c^	12.94 ± 0.72 ^c^	57.54 ± 1.07 ^cd^
	CO50HO50 6	18.97 ± 1.03 ^c^	11.69 ± 2.12 ^c^	59.06 ± 5.23 ^cd^
	CO75HO25 0	28.56 ± 1.65 ^ab^	18.25 ± 2.39 ^b^	57.49 ± 3.13 ^cd^
	CO75HO25 2	29.74 ± 1.23 ^a^	21.65 ± 0.68 ^b^	51.38 ± 3.55 ^d^
	CO75HO25 6	26.61 ± 0.63 ^b^	25.93 ± 1.22 ^a^	35.05 ± 1.52 ^e^
C14:0	CO25HO75 0	4.69 ± 0.02 ^d^	4.14 ± 0.20 ^e^	41.18 ± 2.70 ^a^
	CO25HO75 2	4.63 ± 0.09 ^d^	4.78 ± 0.06 ^e^	31.20 ± 0.50 ^bc^
	CO25HO75 6	4.42 ± 0.20 ^d^	4.01 ± 0.3 ^e^	39.62 ± 2.90 ^a^
	CO50HO50 0	9.03 ± 0.24 ^c^	9.78 ± 0.36 ^cd^	27.79 ± 0.76 ^cd^
	CO50HO50 2	9.36 ± 0.30 ^c^	10.64 ± 0.48 ^c^	24.26 ± 0.98 ^e^
	CO50HO50 6	9.32 ± 0.20 ^c^	9.23 ± 0.41 ^d^	34.01 ± 1.51b ^b^
	CO75HO25 0	15.29 ± 0.57 ^a^	17.31 ± 0.81 ^a^	24.55 ± 0.70 ^de^
	CO75HO25 2	13.90 ± 0.38 ^b^	16.09 ± 0.55 ^b^	22.86 ± 0.52 ^e^
	CO75HO25 6	13.89 ± 0.47 ^b^	16.80 ± 0.66 ^ab^	19.34 ± 0.41 ^f^
C16:0	CO25HO75 0	7.25 ± 0.007 ^e^	9.28 ± 0.10 ^d^	14.70 ± 1.02 ^d^
	CO25HO75 2	7.24 ± 0.35 ^e^	8.18 ± 0.98 ^d^	14.57 ± 0.97 ^d^
	CO25HO75 6	7.005 ± 0.09 ^e^	8.54 ± 0.17 ^e^	18.68 ± 0.54 ^b^
	CO50HO50 0	7.75 ± 0.12 ^d^	10.52 ± 0.17 ^c^	9.49 ± 0.08 ^e^
	CO50HO50 2	7.75 ± 0.25 ^d^	10.04 ± 0.38 ^c^	13.62 ± 0.47 ^d^
	CO50HO50 6	8.13 ± 0.07 ^d^	9.32 ± 0.16 ^d^	23.62 ± 0.60 ^a^
	CO75HO25 0	10.01 ± 0.04 ^a^	12.50 ± 0.09 ^a^	16.75 ± 0.24 ^c^
	CO75HO25 2	8.67 ± 0.04 ^c^	11.27 ± 0.08 ^b^	13.33 ± 1.07 ^d^
	CO75HO25 6	9.31 ± 0.37 ^b^	11.34 ± 0.56 ^b^	18.77 ± 0.73 ^b^
C18:0	CO25HO75 0	3.58 ± 0.14 ^cd^	4.69 ± 0.18 ^bcde^	12.56 ± 0.03 ^c^
	CO25HO75 2	3.58 ± 0.13 ^cd^	4.63 ± 0.23 ^bcde^	13.78 ± 1.04 ^bc^
	CO25HO75 6	3.50 ± 0.04 ^cd^	4.43 ± 0.09 ^cd^	15.69 ± 0.62 ^b^
	CO50HO50 0	3.67 ± 0.13 ^cd^	5.08 ± 0.21 ^bc^	7.76 ± 0.47 ^d^
	CO50HO50 2	3.30 ± 0.02 ^d^	4.24 ± 0.04 ^d^	14.24 ± 0.12 ^bc^
	CO50HO50 6	3.75 ± 0.14 ^bc^	4.47 ± 0.27 ^bcd^	20.59 ± 1.76 ^a^
	CO75HO25 0	4.59 ± 0.37 ^a^	5.94 ± 0.53 ^a^	13.74 ± 0.71 ^bc^
	CO75HO25 2	3.91 ± 0.19 ^bc^	5.13 ± 0.14 ^b^	12.40 ± 1.90 ^c^
	CO75HO25 6	4.15 ± 0.27 ^b^	5.00 ± 0.43 ^bcd^	19.73 ± 1.73 ^a^
C18:1 n-9	CO25HO75 0	12.20 ± 0.22 ^a^	12.49 ± 0.09 ^a^	31.70 ± 0.74 ^bc^
	CO25HO75 2	12.17 ± 0.22 ^a^	11.97 ± 0.47 ^a^	34.40 ± 1.39 ^ab^
	CO25HO75 6	12.06 ± 0.12 ^a^	12.32 ± 0.21 ^ab^	31.85 ± 0.47 ^bc^
	CO50HO50 0	10.56 ± 0.27 ^bc^	11.18 ± 0.40 ^b^	29.44 ± 0.67 ^cd^
	CO50HO50 2	10.00 ± 0.33 ^cd^	9.84 ± 0.54 ^c^	34.37 ± 1.43 ^ab^
	CO50HO50 6	10.92 ± 0.25 ^b^	11.81 ± 0.52 ^ab^	27.92 ± 1.51 ^d^
	CO75HO25 0	10.30 ± 0.57 ^bc^	11.92 ± 0.82 ^ab^	22.89 ± 1.04 ^e^
	CO75HO25 2	9.32 ± 0.34 ^d^	9.29 ± 0.09 ^c^	33.47 ± 3.15 ^ab^
	CO75HO25 6	9.84 ± 0.44 ^cd^	9.29 ± 0.73 ^c^	37.09 ± 2.16 ^a^
C18:2 n-6	CO25HO75 0	42.05 ± 0.07 ^a^	44.43 ± 0.96 ^a^	31.70 ± 0.74 ^cd^
	CO25HO75 2	42.04 ± 0.09 ^a^	42.16 ± 0.30 ^a^	34.40 ± 1.39 ^c^
	CO25HO75 6	42.68 ± 0.60 ^a^	43.66 ± 1.14 ^ab^	31.85 ± 0.47 ^c^
	CO50HO50 0	30.88 ± 0.91 ^b^	33.73 ± 1.55 ^b^	29.44 ± 0.67 ^d^
	CO50HO50 2	28.64 ± 1.38 ^c^	28.98 ± 2.20 ^c^	34.37 ± 1.43 ^c^
	CO50HO50 6	30.91 ± 0.87 ^b^	34.24 ± 1.67 ^b^	27.92 ± 1.51 ^d^
	CO75HO25 0	18.25 ± 1.07 ^e^	20.24 ± 1.47 ^d^	22.89 ± 1.04 ^d^
	CO75HO25 2	18.28 ± 0.73 ^e^	16.96 ± 0.40 ^e^	33.47 ± 3.15 ^b^
	CO75HO25 6	20.30 ± 0.74 ^d^	14.67 ± 1.27 ^e^	51.87 ± 2.40 ^a^
C18:3 n-3	CO25HO75 0	12.66 ± 0.03 ^a^	12.66 ± 0.03 ^a^	26.00 ± 1.34 ^e^
	CO25HO75 2	12.42 ± 0.26 ^a^	12.20 ± 0.53 ^b^	34.53 ± 1.47 ^bc^
	CO25HO75 6	12.57 ± 0.16 ^a^	13.66 ± 0.26 ^a^	27.54 ± 0.46 ^de^
	CO50HO50 0	8.94 ± 0.46 ^b^	9.83 ± 0.78 ^c^	26.71 ± 2.03 ^de^
	CO50HO50 2	8.11 ± 0.34 ^c^	7.07 ± 0.55 ^e^	41.92 ± 2.05 ^ab^
	CO50HO50 6	8.77 ± 0.22 ^b^	8.68 ± 0.46 ^d^	34.04 ± 1.79 ^bc^
	CO75HO25 0	4.81 ± 0.36 ^e^	5.14 ± 0.48 ^f^	28.81 ± 1.32 ^de^
	CO75HO25 2	5.22 ± 0.24 ^de^	5.44 ± 0.09 ^f^	30.45 ± 1.98 ^cd^
	CO75HO25 6	5.83 ± 0.24 ^d^	5.02 ± 0.42 ^f^	42.67 ± 2.45 ^a^

Determined data are presented as mean values followed by the standard deviation (±SD). The different lowercase letters in columns (for each fatty acid separately) indicate significantly different values (*p* ≤ 0.05).

## Data Availability

The original contributions presented in the study are included in the article material, further inquiries can be directed to the corresponding author.
